# UV-Resonance Raman Spectra of Systems in Complex Environments: A Multiscale Modeling Applied to Doxorubicin Intercalated into DNA

**DOI:** 10.1021/acs.jcim.2c01495

**Published:** 2023-02-06

**Authors:** Sara Gómez, Piero Lafiosca, Franco Egidi, Tommaso Giovannini, Chiara Cappelli

**Affiliations:** † Scuola Normale Superiore, Classe di Scienze, Piazza dei Cavalieri 7, 56126 Pisa, Italy; ‡ Software for Chemistry and Materials BV, De Boelelaan 1083, 1081 HV Amsterdam, The Netherlands

## Abstract

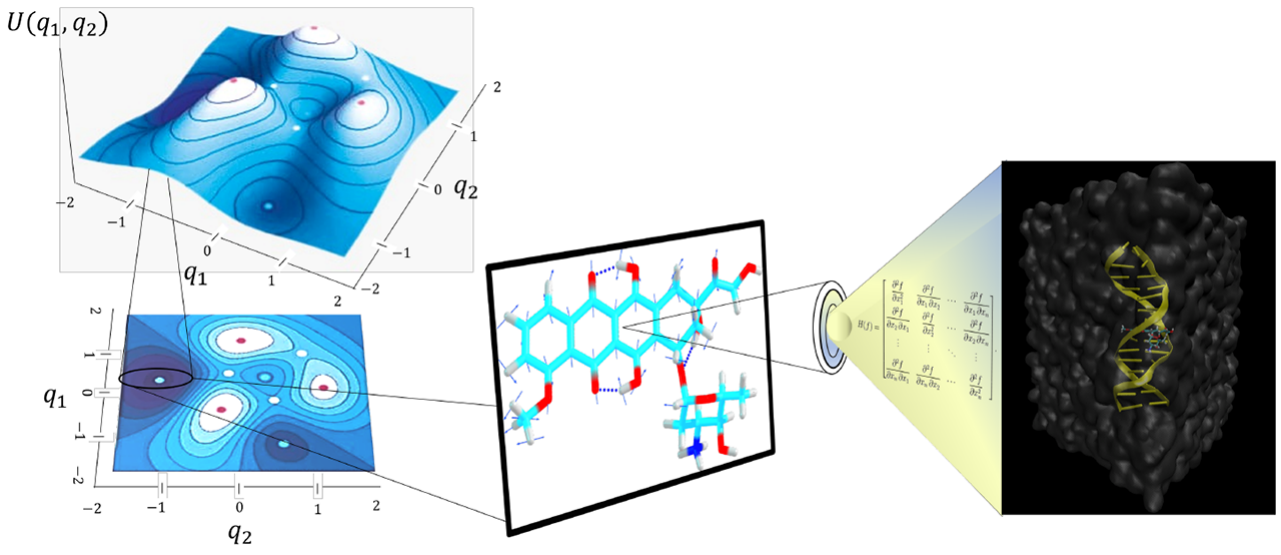

UV-Resonance Raman (RR) spectroscopy is a valuable tool to study the binding of drugs to biomolecular receptors. The extraction of information at the molecular level from experimental RR spectra is made much easier and more complete thanks to the use of computational approaches, specifically tuned to deal with the complexity of the supramolecular system. In this paper, we propose a protocol to simulate RR spectra of complex systems at different levels of sophistication, by exploiting a quantum mechanics/molecular mechanics (QM/MM) approach. The approach is challenged to investigate RR spectra of a widely used chemotherapy drug, doxorubicin (DOX) intercalated into a DNA double strand. The computed results show good agreement with experimental data, thus confirming the reliability of the computational protocol.

## 1 Introduction

UV-Resonance Raman (RR) spectroscopy is among the most powerful techniques used to investigate biological systems.^1^ RR spectroscopy exploits the fact that during Raman measurements the incident frequency is tuned into an electronic absorption band, enhancing selected vibrational modes.^2^ RR offers a unique selectivity as well as a high sensitivity to experimentally detect even traces of compounds, and thus, it finds analytical applications in agriculture, life sciences, explosive detection, art, archeology, and forensics, with additional current research in carbon nanotubes.^3^ The key ingredient in the simulation of RR spectra of isolated systems is the transition polarizability tensor,^4−7^ which can be obtained by exploiting a variety of approaches.^8−22^

When the system under investigation is in solution, the complexity of modeling increases, and quantum mechanics/molecular mechanics (QM/MM) methods have been proven to be particularly successful, thanks to robust computational protocols developed in recent years.^23−26^ Furthermore, in such systems, the partitioning between the QM and MM portions is generally straightforward because no covalent boundary exists between the solute and the solvent. Evidently, if the complexity of the system further increases (e.g., in the case of heterogeneous systems), the existing protocols for isolated or even solvated molecules must be adapted so that the new features can be properly considered in a physically consistent way.

In this work, we extend a computational protocol, recently developed by us, which has been established as the state-of-the-art for the simulation of RR^26−28^ and other diverse spectral signals in aqueous solutions, to target molecules embedded in biological matrices. The protocol involves a series of steps that start with a configurational sampling, and from this, a number of structures are retrieved and used in subsequent quantum-classical calculations.^23,25^ For systems in solution, basically, all kinds of spectroscopies have been covered in recently reported protocols,^23,29,30^ but when it comes to complex environments such as proteins, DNA, and membranes, simulations usually focus on purely electronic spectroscopies.^31,32^

Given the combined electronic-vibrational nature of the RR signal, computing the property in a complex environment requires that the effects arising from electronic and vibrational parts be coherently inserted into the model. Accordingly, electronic transitions, normal modes, and polarizabilities are all ingredients that are influenced by the environment.

Indeed, the computational costs associated with the calculation of the vibrational responses of a complex system, such as a biological matrix, can be prohibitive. This is due to the fact that treating large systems implies including hundreds of vibrations in the calculation of the final spectra. Also, it is worth pointing out that, in order to obtain a reliable spectroscopic signal, the configurational phase space of the target environment system needs to be adequately sampled. This is usually done by resorting to a set of uncorrelated snapshots extracted from molecular dynamics (MD) simulations. However, when dealing with large, complex systems, this means that the vibrational analysis needs to be performed on each configuration, thus further increasing the computational complexity.

**Figure 1:**
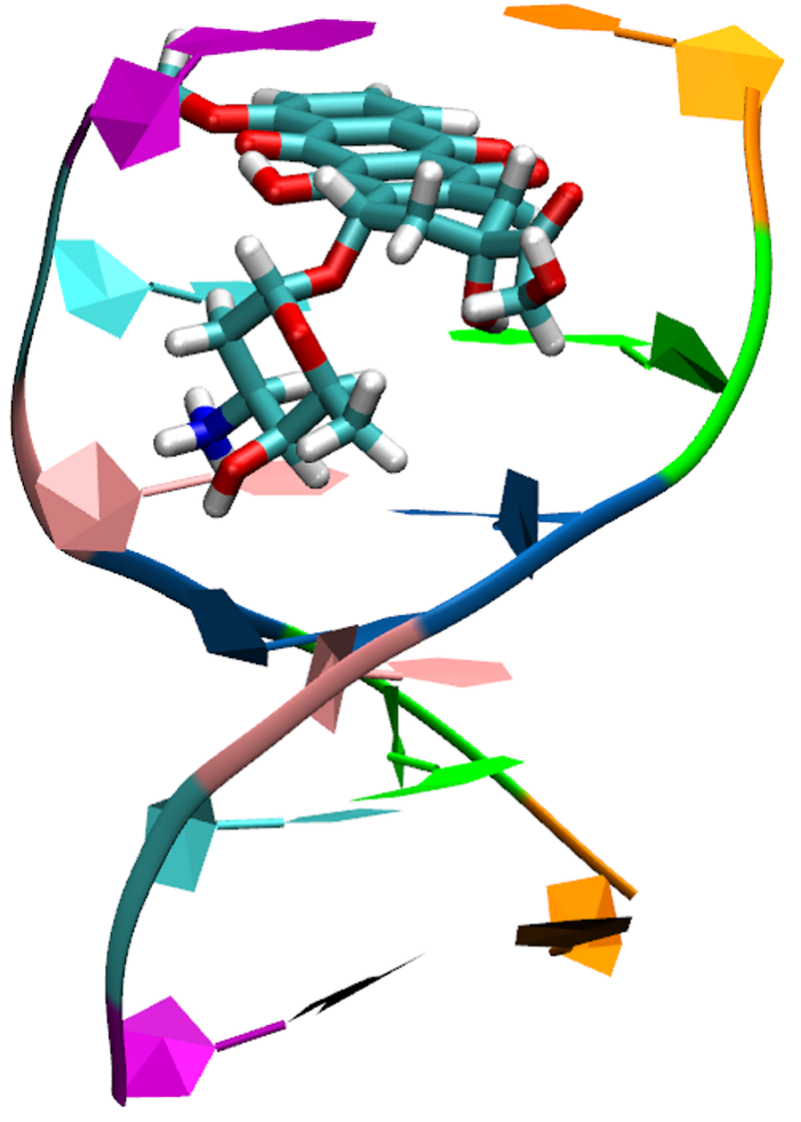
Graphical representation of doxorubicin bound to a d(CGATCG) sequence of DNA.

Here, we propose a series of strategies to compute the normal modes aiming at circumventing this problem. We apply the resulting protocol to doxorubicin (DOX, trade name Adriamycin) intercalated into DNA. DOX is part of the anthracycline anticancer group, and it has been claimed that this drug binds with DNA via an intercalation mechanism.^33,34^Figure 1 displays an intercalation complex between DOX and DNA. Drug/DNA complexes have been computationally studied, and some works^35−47^ are listed in Table S1 in the Supporting Information (SI).

Spectroscopy, and RR in particular, has been crucial for studying DOX in various environments, including DNA. Table S2 in the SI reports important contributions with a variety of techniques.^48−62^ Indeed, many authors have pointed out spectroscopic consequences upon intercalation in the cases of absorption, fluorescence, Raman, and remarkably RR spectra.^51,56^

The paper is organized as follows: first, the methodology and a hierarchy of methods to compute RR in complex environments are described along with computational details. Then, the different approaches are validated for the DOX/DNA systems or DOX dissolved in aqueous solution, by comparison with experimental results. Finally, conclusions and perspectives are drawn.

## 2 Methods

### 2.1 Computational Protocol

**Figure 2:**
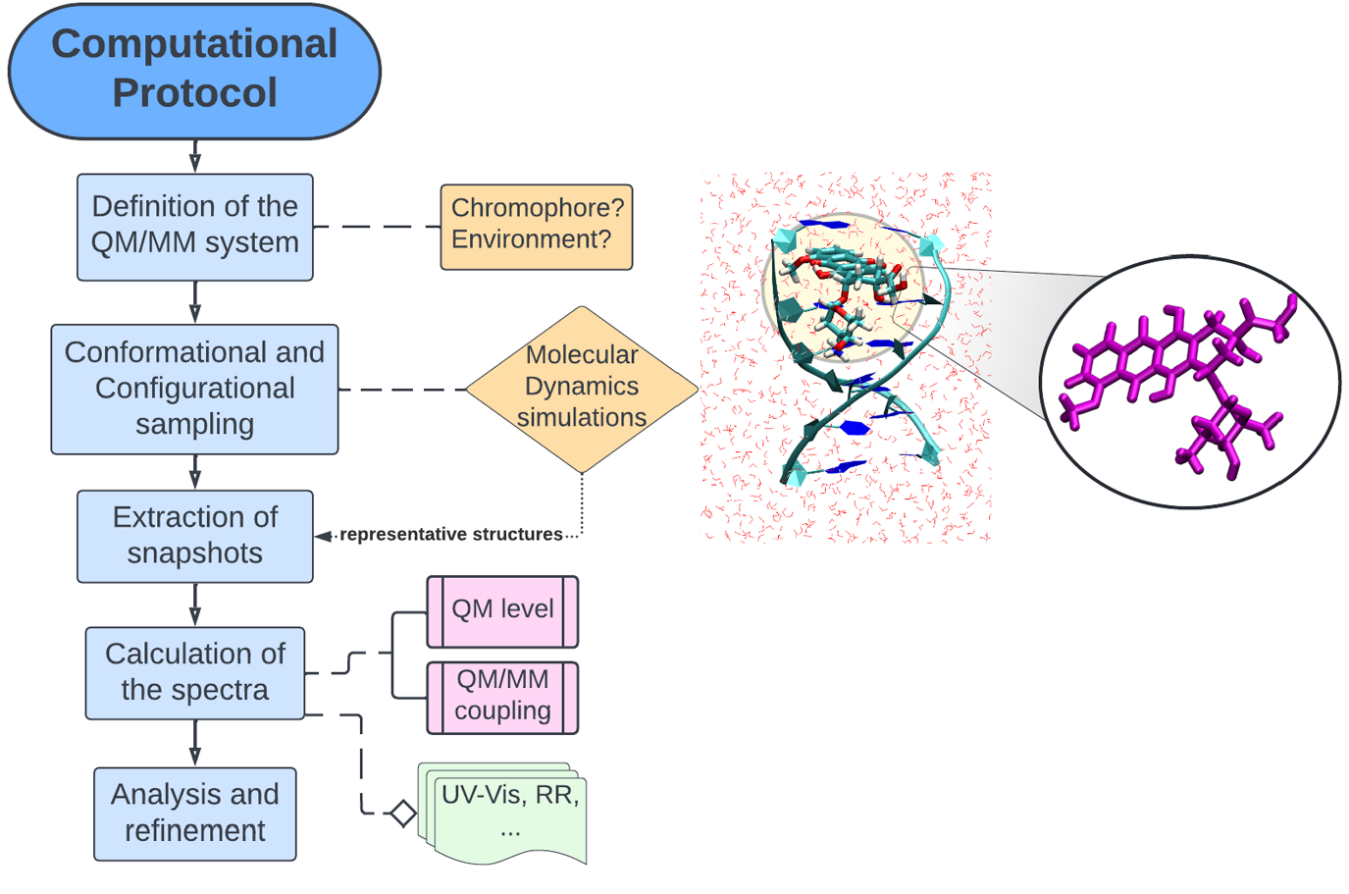
Flowchart of the protocol proposed in this work to model RR spectra of complex systems.

In order to calculate the RR spectra of DOX dissolved in water and intercalated into DNA, we adapt an established approach proposed for aqueous solutions.^23,25^ The protocol we propose is depicted step-by-step in Figure 2. A two-layer QM/MM system is defined for DOX (QM) in water (MM), whereas for the presence of DNA, we consider DOX as the target system (QM) and DNA/Water as the environment (MM) unless otherwise stated. To sample the phase space, we run a classical MD simulation for DOX in water using the same atom types and restrained electrostatic potential (RESP)-derived atomic charges as in ref (44). For the 1:1 DOX/DNA complex, we take the trajectories reported by Jawad et al..^44^ We select the sequence M3 d(CGATCG) because it is one of the strongest binding hexameric sequences of DOX after running binding free energy (BFE) calculations by using MM-PBSA or MM-GBSA methods which gave BFE values of −12.74 and −9.10 kcal/mol, respectively.^44^ From MD runs, 150 and 200 uncorrelated snapshots are taken for DOX/Water (one every 40 ps) and DOX/DNA/Water (one every 30 ps), respectively. Such snapshots contain free DOX or the intercalated DOX/DNA complex with surrounding water molecules within a cutoff distances of 18 and 8 Å, respectively (a selected snapshot is shown in the inset of Figure 2). To reduce the computational cost, clustering analysis of the MD runs is performed by following the methodology proposed in ref (63). Here, 10 and 6 structural families are identified from the trajectories in water and in DNA, respectively. Finally, on the representative structures, we computed RR spectra at the QM/MM level by resorting to both electrostatic (EE)^64,65^ and polarizable embedding (PE) schemes,^23,25,66^ the latter based on the fluctuating charges (FQ) force field.^67^ In particular, while in EE fixed charges are assigned to MM atoms^68^ (equal to those used in the MD runs), in FQ the charges may vary as a consequence of the interaction with the QM density (and vice versa), thus introducing a mutual target-embedding polarization. Notice that in QM/FQ calculations, the QM region consists of the couple DOX/DNA, and the FQ region comprises all water molecules (described by using the parameters of ref (69)). This combination is referred to as QM/QM_DNA_/FQ in what follows.

RR spectra are calculated by resorting to a short-time dynamics approximation. RR intensities are directly computed from the geometrical derivatives of the frequency-dependent polarizability with respect to the normal coordinates.^18−21^ The main advantage of that strategy is that all the electronic states are included in the polarizability, being also well suited for dealing with large molecules or small molecules in complex environments.^18,22^ We investigate different strategies to obtain DOX normal modes, by considering the variants listed in Table 1 and explained in the following paragraphs.

**Table 1:** Inventory of Different Approaches Employed in Calculation of Normal Modes^a^

		QM/EE				QM/FQ		
Snapshots		A0	PHVA	A1	A2	A0	PHVA	A1
Water	150	√						
	10	√	√	√	√	√	√	√
								
DNA	200	√						
	6	√	√	√	√	√	√	√

a Normal modes serve to perform subsequent displacements and final calculations of polarizability derivatives. See the text for a detailed explanation.

### 2.2 Strategies to Obtain Normal Modes

To explore the effect of the quality of the normal modes on the final RR spectra, we systematically increase the level of sophistication in their acquisition. For this purpose, we try four approaches.

#### 2.2.1 The Roughest Approximation, A0

DOX normal modes are computed for each snapshot extracted from the MD simulation, without performing any optimization of the target system. In this way, DOX conformations are preserved in the complex; however, at the QM/MM level, DOX will not generally lie in its energy minimum. Although this approximation might be rather crude, it can give an initial insight into the vibrational spectral shape if the actual DOX geometry is not too different from the equilibrium one.

#### 2.2.2 Partial Hessian Vibrational Approach, PHVA

Prior to the calculation of the normal modes, for each representative structure, DOX geometry is optimized by keeping water molecules or water and DNA base pairs (BPs) frozen in each reduced-size snapshot. This is consistent with the Partial Hessian Vibrational Approach (PHVA),^70−72^ and DOX vibrational degrees of freedom are separated from those of the environment. Notably, this method preserves the environment in the configurations collected from MD simulations.

#### 2.2.3 Transformation (Rotation) Matrix, A1

DOX normal modes are calculated on a reference structure optimized by using the Conductor Screening Model (COSMO)^73^ to describe environmental effects. The dielectric constants for water and DNA given in ref (74) are used. The two geometries, i.e., the one of the reference DOX and the “distorted” DOX in each frame, can be related to each other by means of a fitting (superimposing) procedure that uses rotations and translations transformations in order to minimize the root-mean-squared deviation (RMSD) between the two lists of coordinates. Following that idea, for each frame, we construct a 3 × 3 transformation matrix providing the best alignment between the DOX isolated optimized structure and DOX geometry in the snapshot. To obtain the matrices, we use the *superpose3d* GitHub Repository^75^ that implements the method outlined in ref (76). Finally, the obtained transformation matrix is applied to the normal modes of the isolated optimized DOX to project them onto the extracted MD frame. The A1 strategy is tested on MD representative structures only.

#### 2.2.4 Modification of Adiabatic Molecular Dynamics Generalized Vertical Hessian Ad-MD–gVH Approach, A2

It is an adaptation of the recently proposed mixed quantum-classical approach for the computation of electronic spectra of molecules characterized by a set of stiff (harmonic) modes and one or few internal large-amplitude (soft) motions.^77^ For each MD snapshot, we take the reduced dimensionality Hessian resulting from projecting out the soft coordinates from the ground state Hessian. This results in a new set of frequencies and normal modes, over which we later perform the corresponding displacements. The definition of flexible coordinates for DOX can be found in Section S3 in the SI. To obtain the reduced Hessian, we use the *FCclasses* code,^78^ version 3.0.

All calculations are conducted by using a modified version of the Amsterdam Modeling Suite (AMS), release 2020.202.^79^ In all cases, optimization (when it applies) and normal modes of DOX are calculated at the DFTB3 level, using the 3ob-3-1 parameter set.^80^ Such normal modes are improved using the *Mode refinement* option^81^ implemented in AMS. RR intensities are calculated via complex polarizability derivatives^18^ at the B3LYP/DZP level with an incident frequency of 2.56 or 2.49 eV (477 or 497 nm) and a lifetime of 500 cm^–1^. To compute the polarizability derivatives, the components of the polarizability tensor are obtained for two structures that have been displaced by 0.001 au in two different directions along a vibrational mode. Details of the equations employed to compute cross sections are described in Section S4 of the SI. Excitation wavelengths for RR are chosen based on DFTB/FQ UV–vis spectra already reported by some of us.^46^ Simulated spectra are generated by convoluting RR peaks with a Lorentzian band shape with a half-width at half maximum of 10 cm^–1^. To obtain the final RR spectra, the resulting individual spectra are averaged. Persistence percentages (i.e., the number of times each structure is sampled along the MD) are employed in the averaging process for those cases using only representative structures.

## 3 Results

### 3.1 MD Results and Choice of the Representative Structures

**Figure 3:**
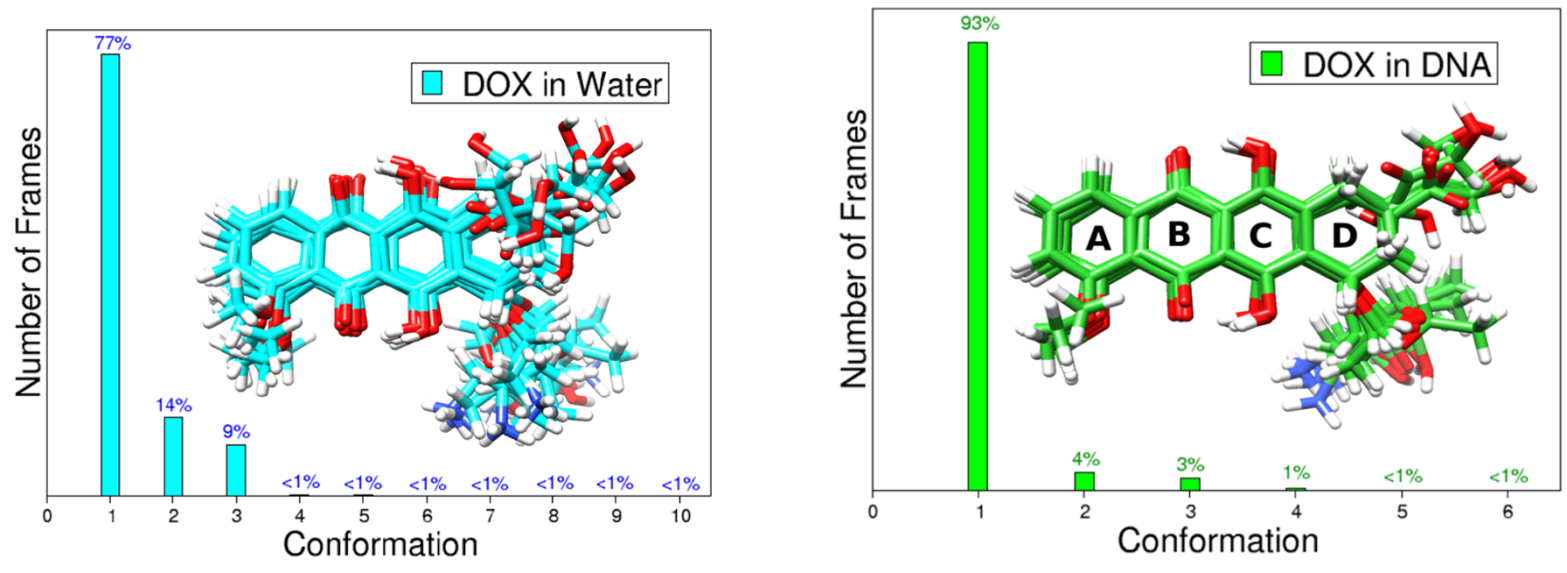
Percentage of frames exhibiting a similar conformation during MD simulations of DOX in water and of DOX intercalated into DNA (Model M3 shown in Figure 1). Differences in geometries for the most sampled conformers are also included as a superimposition.

DOX features three functional domains,^82^ namely, the anthraquinone rings, the anchor, and the daunosamine region which contains an amino sugar group. During MD simulations, the stabilization of the drug via persistent intramolecular HBs between hydroxyl and carbonyl groups in the anthraquinone portion is common to both environments. However, the specificity and energetics of drug/DNA and drug/solvent interactions lead to different scenarios. For the complex formed between DOX and its target, the DNA conformational changes as well as the hydrogen bonding have already been analyzed in previous work.^44^ Summarizing the principal remarks, five HBs between DOX and the chosen DNA sequence are found, namely, two between the O12 (that linked to the ring D as an −OH group, see Figure 3, left) with H atoms bonded to N atoms of the Guanine 8 (G8) and three between the H–N^+^ in the amino sugar and oxygen atoms of the Cytosine 5 (C5) and Thymine 4 (T4). Some of them are highly preserved throughout the simulation. As for DOX in aqueous solution, the general rule is that solute–solvent interactions are dictated by contacts involving O–H and C=O groups with hydrogen and oxygen atoms of the water molecules, in line with previous studies.^83,84^

DOX has many nuclear degrees of freedom and therefore a tremendous conformational diversity. Histograms of the RMSD calculated for each combination of DOX structures found in the simulation (see Figure S2 in the SI) illustrate the flexibility of the DOX moiety along the entire trajectories. It can be seen that there is a wider distribution when DOX is free to move in solution. To group together a few conformers, we resort to a clustering methodology.^63^Figure 3 shows the predominant DOX conformers sampled during both MD simulations. The number of found clusters is 10 (solution) and 6 (DNA), supporting the fact that larger conformational freedom is present for solvated DOX. By looking at the *cluster size*, we see that there is a predominance of two representative isomers, which are expected to contribute more to the calculated property. From the visual inspection of the conformations, it is clear that the anchor domain (glycolaldehyde) and the daunosamine region are the most flexible parts of the system. By observing the superimposition of the structures, the deformation of the last ring is readily appreciable. To apply the strategies explained in the Methods section, we use these sets of conformers. In the PHVA case, those geometries are energy minimized, which primarily changes the values of the dihedral angles involving the external OH groups.

### 3.2 RR Spectra of DOX in Water and in DNA

#### Choice of Incident Frequency

The first necessary step when calculating RR is to set the incident wavelength to irradiate the sample. Early experimental UV–vis of DOX in water places the first electronic transition of the molecule at 480 nm (20,800 cm^–1^)^48^ (477 nm in ref (49)) assigned to a π → π* transition of the quinonoid compound.^49^ According to Angeloni et al.,^51^ a bathochromic shift (≈10 nm) and a hypochromic effect are observed upon complexation with DNA. Computations by Egidi et al.^85^ reveal that the first excited states have a strong charge transfer character involving HOMO → LUMO and HOMO-1 → LUMO transitions. Those molecular orbitals are all localized on the anthraquinone moiety.^86−88^ Using CAM-B3LYP and QM/FQ, the vertical excitation energy for solvated DOX is reported to be 431 nm in ref (84). From simulations using DFTB/FQ, there are some shifts in the calculated spectra with respect to the experimental ones.^46^ By using an electrostatic embedding to treat the environment (see also Figure S3 in the SI), the absorption maximum of DOX intercalated in DNA lies at 497 nm and at 477 nm for DOX in water (B3LYP/DZP level of theory). To correctly reproduce the experimental conditions, we use shifted excitations as incident wavelengths in the simulation of the RR spectra.^16^

#### Analysis of Spectra

**Figure 4:**
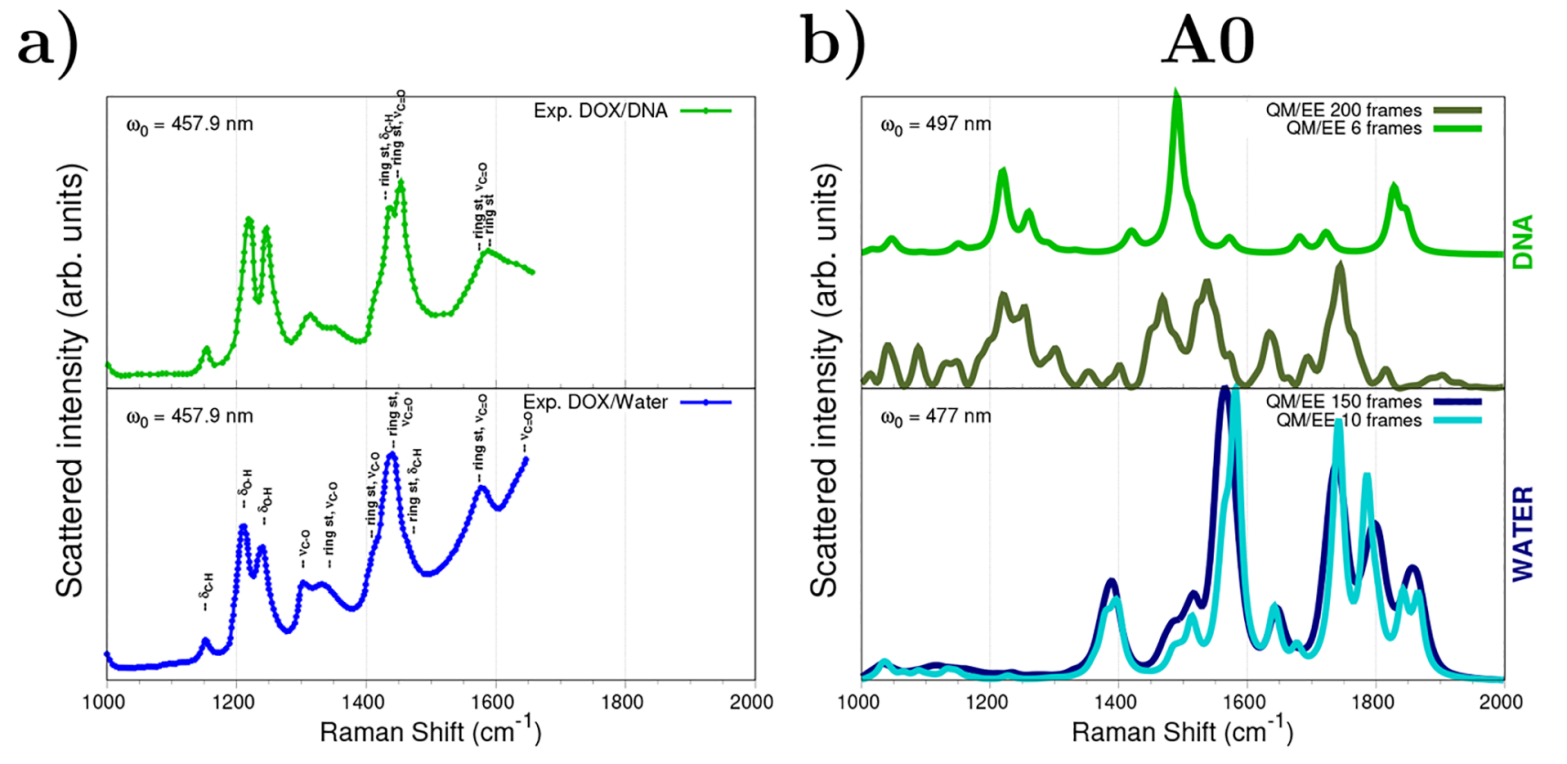
(a) Experimental^51^ resonance Raman spectra of DOX in a DNA solution (top) and in water (bottom). (b) Computed QM/MM EE resonance Raman spectra after applying the approach A0 to displace geometries along the normal modes. RR intensities are calculated through complex polarizability derivatives using a damping factor of 500 cm^–1^. A Lorentzian broadening with an fwhm = 20 cm^–1^ is used. QM level: B3LYP/DZP. All spectra are scaled such that the maximum intensity is unity.

Figure 4 compares simulated QM/EE RR spectra for DOX in water and for the DOX/DNA solution together with the experimental reports by Angeloni et al.^51^ (in the 1000–2000 cm^–1^ region). Assignments of the major bands are given in Figure 4a and can also be found in refs (57 and 59). QM/EE RR spectra are computed by using the whole set of extracted snapshots (200 and 150 frames for aqueous and DNA solutions, respectively) and the representative structures provided by the clustering algorithm (10 and 6 frames for aqueous and DNA solutions, respectively). Although for DOX in water remarkable intensity changes are expected moving from preresonance to resonance conditions,^49^ we point out that we are not interested in simulating enhancement factors, but in constructing a reliable computational tool to model RR spectra and examine the spectral changes when varying the environment.

RR of DOX is dominated by normal modes associated with the condensed aromatic rings, together with the typical signals of hydroxyanthraquinones.^48,49,59,60^ The key point in those findings is that the vibrations of the aromatic ring modes (rings A, B, and C, see right panel of Figure 3) are coupled with the π → π* electronic transition; therefore, it is not surprising that these modes are enhanced in RR spectra. If we compare the two experimental spectra in Figure 4a, we can see that there are some spectral perturbations suggesting a direct interaction between the drug and DNA. At this point of the discussion, it is useful to briefly summarize the effect of complexation on diverse spectroscopies as mentioned by some authors: (1) Besides bathochromic and hypochromic effects on absorption spectra mentioned above and reported to occur upon the formation of the complex between DOX and DNA, there is also a reduction of fluorescence.^51,56,89^ (2) Notable differences between RR spectra of the drug and of the complex are observed in the ranges near 450 cm^–1^ (not shown here) and 1400 cm^–1^. In particular, Angeloni et al.^51^ indicates that the bands at 430 and 1420 cm^–1^, which are very weak for the pure drug, become prominent upon complexation. (3) Yan et al.^56^ report that interactions between DOX and DNA primarily perturb the phenolic group and the π system of the drug. Thus, from RR spectra, it is apparent that once the chromophoric rings are intercalated between adjacent DNA BPs, the intensity of the band at 444 cm^–1^ decreases and turns into a poorly resolved shoulder at 437 cm^–1^ (not shown here), accompanied by the splitting of the 1439 cm^–1^ band into two sub-bands at 1431 and 1449 cm^–1^ associated with the skeletal mode and the CCO stretching mode, respectively. Also, the bands at 1210 and 1241 cm^–1^ shift to 1213 and 1243 cm^–1^, respectively. The latter band becomes sharper and moderately stronger in comparison with the former. Findings from Manfait et al.^50^ and Smulevich et al.^57^ support such variations. (4) Similar prominent spectral changes in the same regions are claimed by Smulevich and Feis^53^ on the basis of SERS experiments.

#### Spectra Obtained with the EE and A0 Schemes

Moving to QM/MM EE spectra presented in Figure 4b and computed under the cheapest approach, A0, i.e., without optimizing the geometries extracted from MD trajectories, we see that for the intercalated DOX the position and shape of most resonance Raman bands are in reasonably good agreement with the experimental observations. In contrast, by comparing computed and experimental RR spectra of aqueous DOX, it is clear that there are very diverse relative intensities and some important bands, as those associated with δ_OH_ (experimentally at around 1215 and 1245 cm^–1^) are completely absent in simulated spectra. This is probably due to the occurrence of imaginary frequencies in both stiff and soft normal modes involving the OH group, which originate from the fact that the structures have not been reoptimized within the A0 approximation. Imaginary frequencies also appear in the complex with DNA, although in a smaller number (15 vs 3 on average as reported in Figure S4 in the SI). Interestingly, this is related to the aforementioned rigidity of the structure when DOX is sandwiched within the DNA via stacking interactions. Furthermore, the out-of-equilibrium conditions yield a strong shift of the ν_C=O_ and ring stretching bands, which are wrongly predicted above 1800 cm^–1^. This again indicates the inappropriateness and unsuitability of the A0 method. It can be finally noticed that RR spectra simulated by using the whole set of snapshots and the representative clustered ones are almost superimposable for DOX in water, while for the DOX/DNA complex some discrepancies, mainly related to relative intensities, are reported. Nevertheless, the main features of RR spectra are reproduced, and the discrepancies are reduced if DNA is included in the QM region in the QM/QM_DNA_/FQ modeling (see also Figures S5 and S6 in the SI).

#### Spectra Obtained with the PHVA Scheme

**Figure 5:**
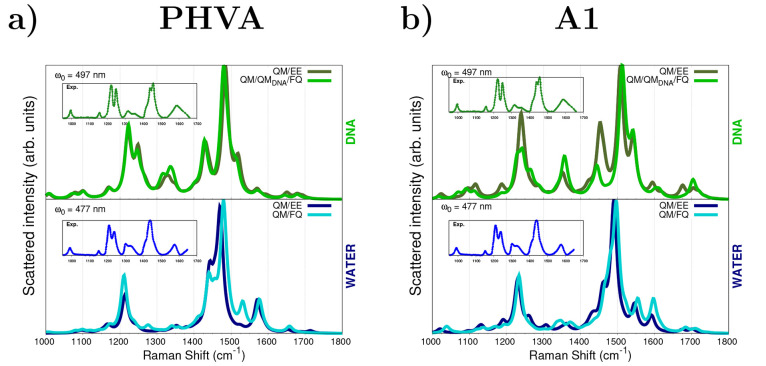
RR spectra of DOX in water and intercalated into DNA. RR intensities are calculated through complex polarizability derivatives using a damping factor of 500 cm^–1^. Results from (a) PHVA and (b) A1 approaches are shown, see text. A Lorentzian broadening with an fwhm = 20 cm^–1^ is used. QM level: B3LYP/DZP. All spectra are scaled such that the maximum intensity is unity. Experimental RR spectra from ref (51) are given in the insets.

We now move to comment on the results obtained by applying the PHVA method to the representative structures, i.e., optimizing the solute in every MD snapshot while freezing the rest of the nuclear coordinates (see Figure 5a). We recall here that when the polarizable FQ scheme is used for the DNA solution, DNA is also included in the QM region, but only the DOX moiety is optimized (QM/QM_DNA_/FQ in Figure 5). Remarkably, PHVA provides a better agreement with the experimental spectra than that found with the A0 approach. Overall, computed frequencies well reflect experimental values, while peaks’ relative intensities exhibit appreciable discrepancies. For instance, the δ_OH_ 2-fold band appears as a single signal accompanied by a shoulder in the computed spectra in water. Furthermore, the broadband measured at about 1600 cm^–1^ is barely visible in DNA. This is due to the fact that the ring stretching modes at 1575 and 1588 cm^–1^ mix to the modes belonging to the 1400–1500 cm^–1^ region, which are assigned to ν_C=C_ + ν_C–C_ vibrations. As a matter of fact, a well-documented slight impact of the drug:DNA ratio^53^ has been reported for such bands and for the ν_C=O···H_ (hydrogen-bonded) vibration,^49,57,59^ which is experimentally visible at around 1644 cm^–1^ (simulated at 1680 cm^–1^).

#### Spectra Obtained with the A1 Scheme

In order to preserve the effects coming from the environment, RR spectra of DOX in water and DNA are computed by using the A1 scheme, which consists in taking the normal modes of one of the lowest energy DOX conformations and applying a rotation matrix (generated from an alignment procedure) to adjust them to the DOX actual geometry in each snapshot. Clearly, this procedure is much cheaper than PHVA, because a single geometry optimization is required. RR spectra computed for the representative structures are presented in Figure 5b. It can be observed that A1 spectra are especially similar to PHVA ones (see previous section) and are also in good agreement with the experiments. Only moderate differences in the position and shape of some bands are reported as compared to PHVA RR spectra (see also Figure 5a). As an example, the bands associated with the ν_C–C_ + ν_C–O_ modes (∼1330 cm^–1^) are enhanced. However, few mismatches in relative intensities are still present, particularly for the most intense peaks, as the δ_OH_, of which the intensity is significantly underestimated by A1 calculations.

As observed in Figure 5, for both approaches, namely, PHVA and A1, RR spectra of the DOX/DNA complex are very similar to those calculated in solution. However, some substantial differences can be appreciated. The band at 1245 cm^–1^ is more enhanced for the DOX/DNA system, and a noticeable splitting of the band at 1441 cm^–1^ is observed. Such findings perfectly reproduce experimental features. Indeed, such bands are related to the two DOX C=O···H–O groups, which are reported^53^ to be involved in the interaction with DNA.

By comparing DOX RR spectra computed by using QM/EE and QM/FQ embedding schemes, it is possible to observe the marginal effects of mutual polarization. The same holds valid for the inclusion of the DNA in the QM region (QM/QM_DNA_/FQ), highlighting the fact that nonelectrostatic interactions between DOX and the DNA basis pairs play minor roles as compared to electrostatics.

#### Spectra Obtained with the A2 scheme

**Figure 6:**
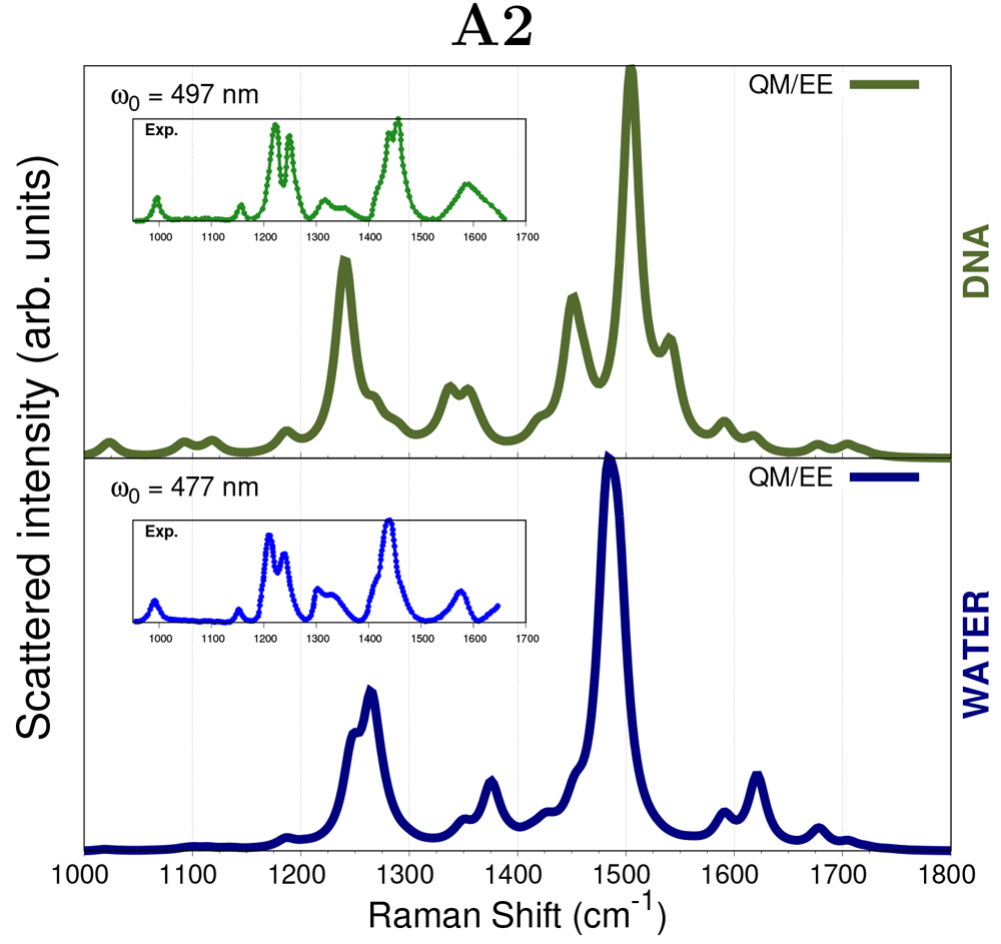
RR spectra of DOX in water and intercalated into DNA. RR intensities are calculated through complex polarizability derivatives using a damping factor of 500 cm^–1^. Results from the A2 approach are shown, see text. A Lorentzian broadening with an fwhm = 20 cm^–1^ is used. QM level: B3LYP/DZP. All spectra are scaled such that the maximum intensity is unity. Experimental RR spectra from ref (51) are given in the insets.

The last proposed approach is based on a modified version of the Ad-MD–gVH approach,^77^ which has originally been designed as a general method for computing electronic spectra of flexible dyes in explicit environments. As explained above, in our adaptation, the DOX Hessian matrix has been constructed by removing all the soft coordinates, which involve the methyl and NH_3_^+^ torsions as well as the CCOH dihedral angle, the latter belonging to the DOX glycolaldehyde portion. We remark that this choice is indeed arbitrary and has been conducted by selecting all the normal modes that were involved in the computed imaginary frequencies. Figure 6 reports A2 RR spectra for DOX in water and into DNA, which are computed at the QM/EE level only due to the observed similarities with more sophisticated approaches. In general, A2 frequencies well resemble the experiments, outperforming the other methods (see Table S3 in the SI). Also, all prominent spectral features are reproduced. This is due to the fact that the normal modes that have been eliminated in the construction of the Hessian matrix are not associated with any enhanced peak in the selected region. Moreover, the DNA complexation basically alters the 1400–1550 cm^–1^ spectral region, and it is also connected to a reduction in the intensity of the ν_C=C_ and ν_C=O···H_ vibrations at 1587 and 1642 cm^–1^. From a comparison between A2 RR spectra and those obtained by resorting to A0, PHVA, and A1 approximations (see also Figures S7 and S8 in the SI), a small improvement is observed, regarding the enhancement of the peaks at 1308 and 1345 cm^–1^ and the splitting of the peaks at 1215 and 1245 cm^–1^ in the aqueous solution. On the other hand, differently from the other approximations, such bands are mixed together when DOX interacts with DNA.

#### Continuum Modeling

For the sake of completeness, we report RR spectra of DOX in water and DNA by describing environmental effects with an implicit, continuum description, obtained by means of the COSMO model (see Figure S9 in the SI). As it has also been reported in previous studies,^74^ a poor agreement with the experimental trends is obtained, as can be noticed in the largely overestimated intensity of the peaks located in the 1300–1400 cm^–1^ region. This further demonstrates the benefit of our atomistic modeling of the environment.

## 4 Conclusions and Final Remarks

In this work, we have proposed and analyzed different computational protocols for modeling RR spectra of doxorubicin dissolved in aqueous solution and intercalated into DNA. The models are based on a multiscale QM/MM approach, which possibly accounts for mutual QM/MM polarization effects. RR spectra have been calculated by numerically differentiating the frequency-dependent complex polarizability with respect to normal vibrational coordinates. To correctly account for the configurational variability of the systems, several snapshots from MD trajectories have been extracted and classified into different structural families based on the clustering analysis proposed in ref (63). RR spectra have subsequently been calculated for representative snapshots of each group. To obtain normal modes and proceed with geometry displacements, four approaches have been tested: (i) A0, i.e., the computationally cheapest one, involves the calculation of normal modes on raw, unoptimized, structures—by definition configurations out of equilibrium—and that, as expected, yields a poor reproduction of experimental values. (ii) Calculating the normal modes from optimized DOX structures by applying the PHVA approach, which improved the results with respect to A0. (iii) Avoiding the calculation of normal modes for each snapshot by employing those of isolated DOX and then using a linear transformation—different from one snapshot to another—to project them onto the actual DOX structure in each snapshot. This method yields computed values in good agreement with experimental observations while preserving the effects of the environment. Lastly, (iv) by borrowing the idea that flexible coordinates can be separated from stiff ones, in each representative snapshot along the MD,^77^ normal modes are recomputed from a reduced dimensionality Hessian. This last approach gives results in good agreement with experimental findings but requires a prescreening procedure to analyze the occurrence of imaginary frequencies and to determine whether they can be reduced by moving such soft coordinates to what Cerezo et al.^77^ called the “classical set”. On the whole, the results reported in this paper show that reliable RR spectra of doxorubicin in complex environments, treated atomistically, are obtained, in satisfactory agreement with experimental data. To conclude, it is amply documented that the specific DNA sequence plays an important role in the binding of a ligand to DNA strands. In that respect, the proposed methodology is general enough to treat different sequences and types of DNA binders, which opens up the possibility of computationally screening different drug candidates for any selected binding site. Finally, for large systems such as the one analyzed in this work, it would be interesting to compare in future studies our RR results with data computed with full vibronic approaches, such as those that have been proposed by some of us for the simplest cases.^9,16^

## Data Availability

DOX structure has been optimized by using the AMS code (version 2020.202 http://www.scm.com). MD simulations of DOX in aqueous solutions have been performed by using Gromacs version 2020.3 (https://www.gromacs.org). Structures of DOX intercalated into DNA have been provided by Professor Wai-Yim Ching and his research group at University of Missouri-Kansas City. QM/MM calculations have been performed by using a modified version of AMS release 2020.202 (http://www.scm.com).

## Supporting Information Available

Inventory of experimental and computational works on DOX and DOX/DNA complexes, definition of flexible coordinates, equations for RR intensities, RMSD distributions, and additional spectra (PDF)
