# Supplementary material for: UV-Resonance Raman Spectra of Systems in Complex Environments: A Multiscale Modeling Applied to Doxorubicin Intercalated into DNA
Source: J Chem Inf Model. 2023 Feb 6;63(4):1208–17. doi: 10.1021/acs.jcim.2c01495 (PMC9976284; doi:10.1021/acs.jcim.2c01495)
Supplement: Supplementary file 1 — ci2c01495_si_001.pdf [file ci2c01495_si_001.pdf]

UV-Resonance Raman Spectra of Systems in Complex Environments: A Multiscale Modeling applied to Doxorubicin intercalated into DNA

Sara Gómez,[†] Piero Lafiosca,[†] Franco Egidi,[¶] Tommaso Giovannini,[†] and Chiara Cappelli,[†]

[†]*Scuola Normale Superiore, Classe di Scienze, Piazza dei Cavalieri 7, 56126, Pisa, Italy*
[¶]*Software for Chemistry & Materials BV, De Boelelaan 1083, 1081 HV Amsterdam, The Netherlands*

Correspondence: sara.gomezmaya@sns.it, chiara.cappelli@sns.it

Supporting information

Contents

S1 Some computational works on DOX-DNA complexes **S2**

S2 Spectral measurements available for DOX **S3**

S3 Definition of flexible coordinates **S4**

S4 Equations to calculate RR intensities **S5**

S5 Further MD results **S6**

S6 Additional spectra and comparisons **S7**

S1 Some computational works on DOX-DNA complexes

Table S1: Survey of the different computational works performed on doxorubicin-DNA complexes

Year	Reference	Topic
1980	[1]	Intercalation of doxorubicin with dinucleotide dimers
2008	[2]	Intercalation of Daunomycin into Stacked DNA Base Pairs
2010	[3]	Conformational diversity of anthracycline anticancer antibiotics
2012	[4]	MD simulations for intercalation of DOX to DNA fragments
2014	[5]	MD simulations for the interaction between DOX and decanucleotide models
2015	[6]	Electronic structure of DOX in different molecular environments: isolated, solvated, and intercalated in a DNA complex
2005	[7]	Self-aggregated drugs in aqueous solution and
2008	[8]	drug–DNA complexes using restrained molecular
2009	[9]	dynamics (RMD) with constrained H–H distances
2019	[10]	MD study for DOX with two hexadecamer dsDNA sequences and binding free energy (BFE) calculations
2020	[11]	MD study for DOX with sixteen different tetradecamer dsDNA sequences and binding free energy (BFE) calculations
2021	[12]	DFTB/FQ absorption spectra of DOX/water/DNA
2021	[13]	Molecular docking for DOX with B-DNA dodecamer and DNA hexamer

S2 Spectral measurements available for DOX

Table S2: Experimental results[d] available for Doxorubicin

Authors and year	UV–Vis[a]		FT–IR		Raman		RR		SER(R)S	
	H_2O	H_2O–DNA	H_2O	H_2O–DNA	H_2O	H_2O–DNA	H_2O	H_2O–DNA	H_2O	H_2O–DNA
Hillig and Morris [14], 1976					✓<br>632.8 nm					
Manfait et al. [15], 1981	✓				✓<br>615 nm		✓<br>457.9 nm			
Manfait et al. [16], 1982	✓	✓						✓<br>457.9 nm		
Angeloni et al. [17], 1982	✓	✓					✓	✓<br>457.9 nm		
Manfait and Theophanides [18], 1983				✓						
Smulevich and Feis [19], 1986									✓<br>457.9 nm	✓
Nonaka et al. [20], 1990							✓<br>457.9 nm		✓<br>488 nm	
Beljebbar et al. [21], 1995									✓<br>514.5 nm	✓
Yan et al. [22], 1997							✓<br>476.5 nm (300–550 cm^{-1})<br>457.9 nm (1150–1650 cm^{-1})	✓	✓<br>457.9 nm	✓
Smulevich et al. [23], 2001[b]	✓	✓					✓<br>457.9 nm	✓		
Lee et al. [24], 2004	✓	✓						✓<br>257 nm	✓	✓
Das et al. [25], 2010[c]			✓		✓<br>830 nm		✓<br>457 nm			
Szafraniec et al. [26], 2016	✓		✓		✓<br>785 nm		✓<br>488 nm			

[a]Some authors claimed that RR spectrum of DOX (488 nm excitation wavelength) in water solution is dominated by fluorescence and the signals are hardly seen.[26]
[b]EPI
[c]Raman Measurements are available also for solid samples
[d]Other studies include fluorescence[27]

S3 Definition of flexible coordinates

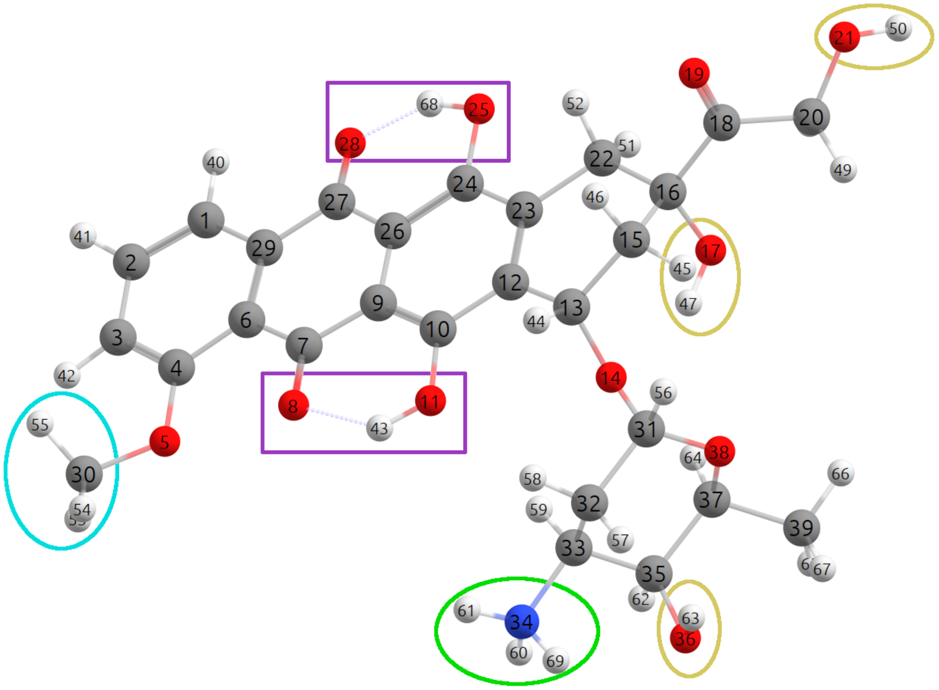

Figure S1: Molecular structure of Doxorubicin. The numbering of atoms used to define the relevant ICs is shown.

The methyl and ammonium group rotations are defined (see Figure S1 for labeling) as

$$\delta_{\mathrm{CH_3}} = d(53, 30, 5, 4) + d(54, 30, 5, 4) + d(55, 30, 5, 4) \tag{S1}$$

$$\begin{aligned} \delta_{\mathrm{NH_3^+}} = & d(60, 34, 33, 59) + d(61, 34, 33, 59) + d(69, 34, 33, 59) \\ & d(60, 34, 33, 35) + d(61, 34, 33, 35) + d(69, 34, 33, 35) \end{aligned} \tag{S2}$$

In the case of the hydroxyl groups, the torsions to be removed are defined as

$$\delta_1 = d(63, 36, 35, 33) + d(63, 36, 35, 37) + d(63, 36, 35, 62) \tag{S3}$$

$$\delta_2 = d(47, 17, 16, 22) + d(47, 17, 16, 15) + d(47, 17, 16, 18) \tag{S4}$$

$$\delta_3 = d(50, 21, 20, 48) + d(50, 21, 20, 49) + d(50, 21, 20, 18) \tag{S5}$$

For torsions involving O11–H43 and O25–H68 hydroxyl groups, we did not remove any internal coordinates since those groups are participating in the enhancements that lead to the final RR spectra.

S4 Equations to calculate RR intensities

The spontaneous Raman scattering cross-section is usually calculated at the DFT level using response theory by differentiating the complex polarizability with respect to the normal mode displacements, calculated for a perturbation with angular frequency ω corresponding to the one of the light source. Given the vibrational transition polarizability α^i corresponding to an excitation of the i-th normal mode, then the cross-section σ_i can be expressed in terms of the Raman rotational invariants:

$$a_i^2 = \frac{1}{9}\sum_{ab} \alpha^*_{aa,i}\alpha_{bb,i} = \frac{1}{9}|\alpha_{xx,i} + \alpha_{yy,i} + \alpha_{zz,i}|^2 \tag{S6}$$

$$g_i^2 = \frac{1}{2}\sum_{ab}(3\alpha^*_{ab,i}\alpha_{ab,i} - \alpha^{i*}_{aa,i}\alpha_{bb,i}) \tag{S7}$$

$$\sigma_i = \left(\frac{\omega - \omega_i}{c}\right)^4 \frac{45a_i^2 + 7g_i^2}{45} \tag{S8}$$

S5 Further MD results

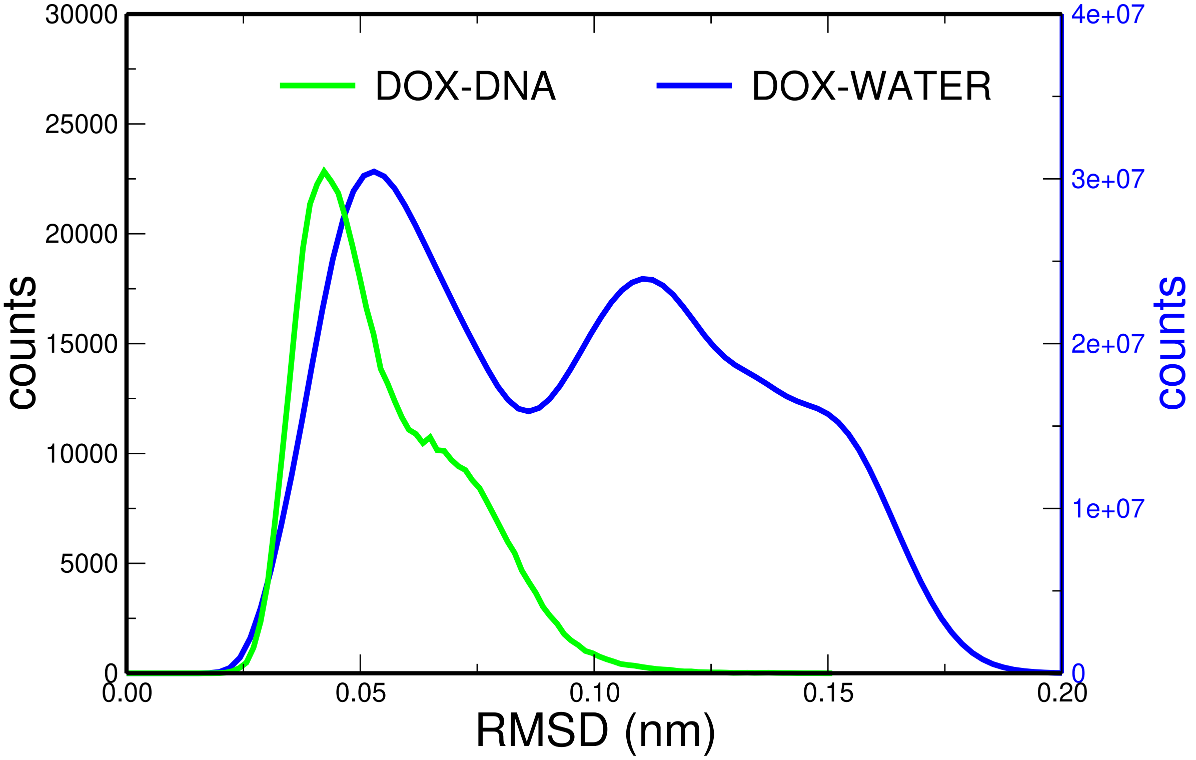

Figure S2: Distribution of the Root-Mean-Square Deviation (RMSD) for each combination of DOX structures along the MD trajectories

S6 Additional spectra and comparisons

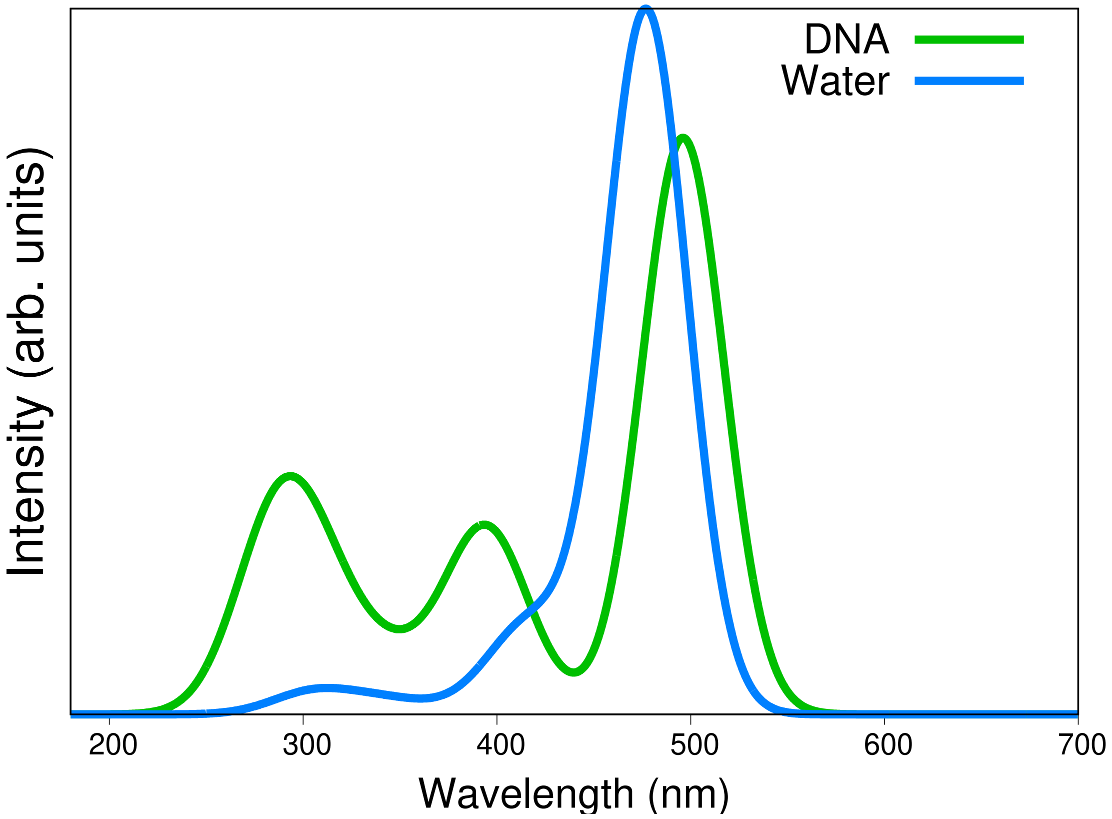

Figure S3: UV-Vis spectra of DOX in water and in DNA solution

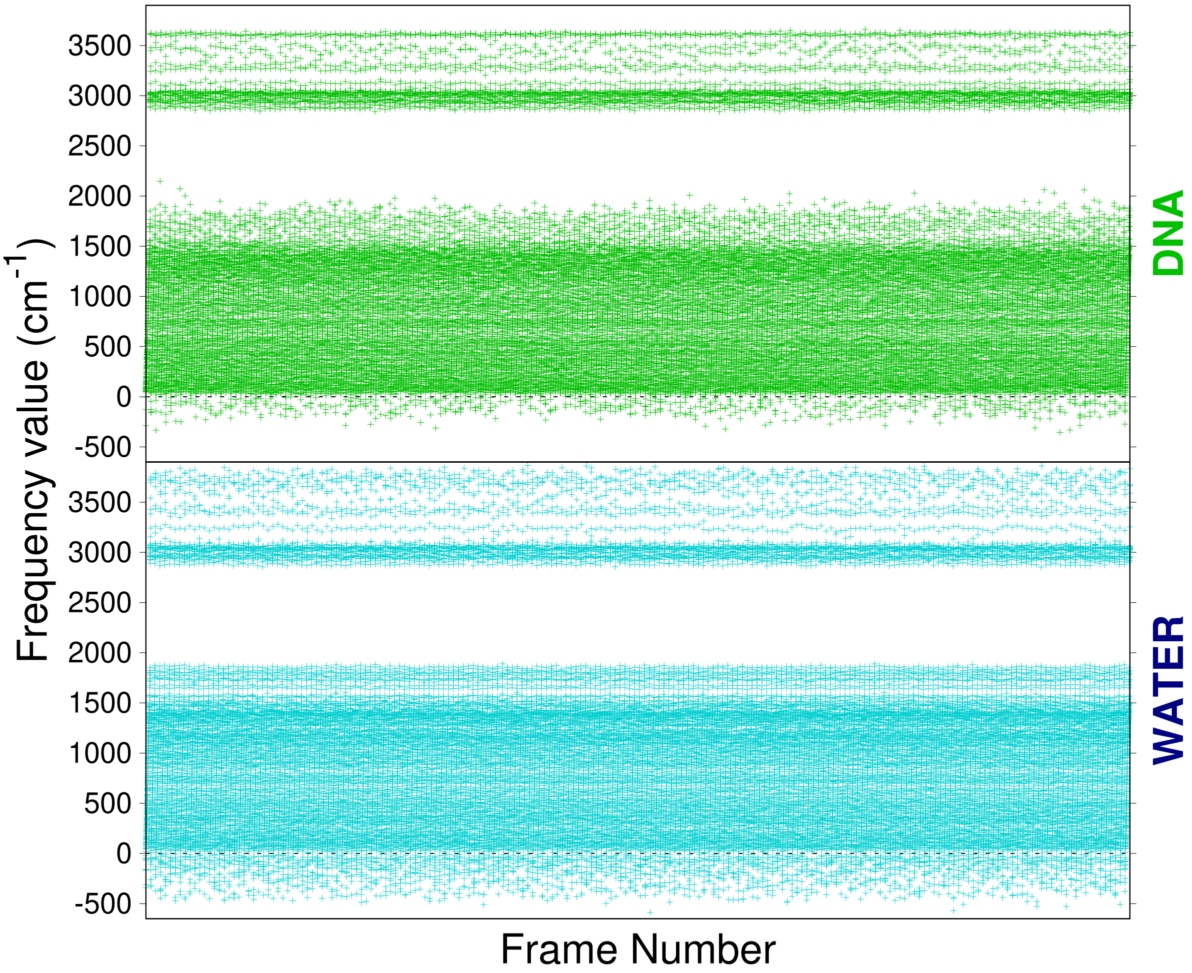

Figure S4: Ranges for frequencies when the DOX normal modes are directly calculated on the snapshots coming from the MD trajectory (A0 strategy)

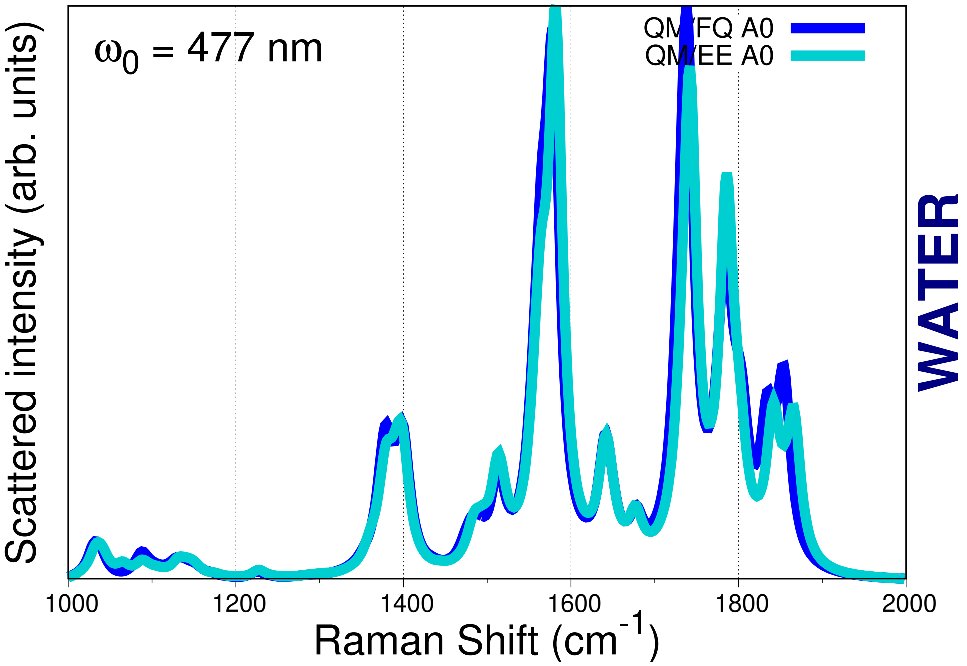

Figure S5: Comparison between QM/EE and QM/FQ RR spectra of DOX in Water, after applying the A0 strategy on the ten representative structures

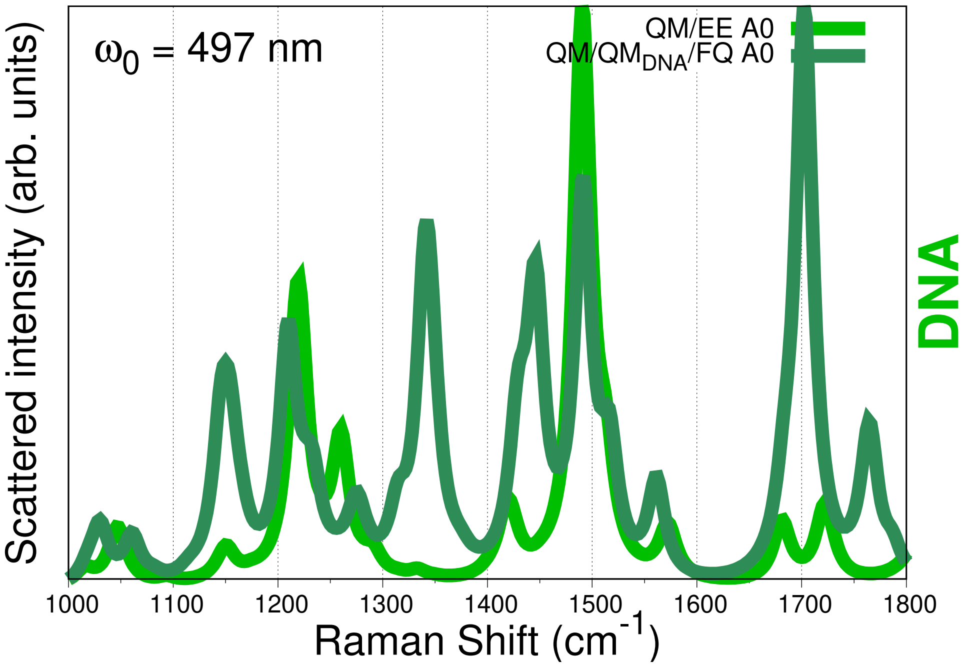

Figure S6: Comparison between QM/EE and QM/FQ RR spectra of DOX in DNA solutions, after applying the A0 strategy on the six representative structures

	Experiment		QM/EE				QM/FQ		
	Position	Assignment	A0	PHVA	A1	A2	A0	PHVA	A1
Water	1153	δ_{C-H}	1140 (-1%)	1170 (1%)	1190 (3%)	1180 (2%)	1140 (-1%)	1175 (2%)	1160 (1%)
	1210	δ_{O-H}	A (-100%)	1215 (0%)	1218 (1%)	1240 (2%)	A (-100%)	1210 (0%)	1220 (1%)
	1241	δ_{O-H}	A (-100%)	1215 (-2%)	1218 (-2%)	1260 (2%)	A (-100%)	1210 (-2%)	1220 (-2%)
	1303	ν_{C-O}	1380 (6%)	A (-100%)	1303 (0%)	1350 (4%)	1380 (6%)	1318 (1%)	1320 (1%)
	1343	ring st, ν_{C-O}	1390 (3%)	1360 (1%)	1370 (2%)	1370 (2%)	1390 (3%)	1330 (-1%)	1370 (2%)
	1412	ring st, ν_{C-O}	1500 (6%)	1440 (2%)	1470 (4%)	1450 (3%)	1500 (6%)	1425 (1%)	1450 (3%)
		ring st, δ_{C-H}							
	1439	ring st, $\nu_{C=O}$	1560 (8%)	1460 (1%)	1480 (3%)	1485 (3%)	1550 (8%)	1470 (2%)	1480 (3%)
	1474	ring st, δ_{C-H}	1625 (10%)	A (-100%)	1500 (2%)	1505 (2%)	1625 (10%)	1525 (3%)	1509 (2%)
	1575	ring st, $\nu_{C=O}$	1710 (9%)	1572 (0%)	1540 (-2%)	1590 (1%)	1708 (8%)	1575 (0%)	1550 (-2%)
		ring st				1610 (2%)			1595 (1%)
	1645	$\nu_{C=O}$	1830 (11%)	1660 (1%)	1680 (2%)	1675 (2%)	1830 (11%)	1660 (1%)	1680 (2%)
DNA	1153	δ_{C-H}	1160 (1%)	1180 (2%)	1190 (3%)	1180 (2%)	1160 (1%)	1180 (2%)	1180 (2%)
	1213	δ_{O-H}	1215 (0%)	1215 (0%)	1230 (1%)	1240 (2%)	1210 (0%)	1215 (0%)	1218 (0%)
	1244	δ_{O-H}	1230 (-1%)	1240 (0%)	1230 (-1%)	1260 (1%)	1270 (2%)	1240 (0%)	1260 (1%)
	1302	ν_{C-O}	1300 (0%)	1315 (1%)	1350 (4%)	1315 (1%)	1320 (1%)	1307 (0%)	1345 (3%)
	1339	ring st, ν_{C-O}	1325 (-1%)	1350 (1%)	1350 (1%)	1328 (-1%)	1350 (1%)	1320 (-1%)	1350 (1%)
	1431	ring st, δ_{C-H}	1440 (1%)	1430 (0%)	1450 (1%)	1450 (1%)	1440 (1%)	1428 (0%)	1440 (1%)
	1449	ring st, $\nu_{C=O}$	1520 (5%)	1480 (2%)	1505 (4%)	1505 (4%)	1485 (2%)	1470 (1%)	1507 (4%)
	1575	ring st, $\nu_{C=O}$	1620 (3%)	1575 (0%)	1590 (1%)	1590 (1%)	1560 (1%)	1575 (0%)	1595 (1%)
	1588	ring st	1725 (9%)			1610 (1%)	1700 (7%)		
		$\nu_{C=O}$	1810 (–%)	1660 (5%)	1675 (5%)	1675 (–%)	1810 (–%)	1660 (5%)	1680 (6%)

Table S3: Performance of the different A0, PHVA, A1, and A2 approaches when reproducing experimental peak positions in RR spectra of DOX in water and DNA. See the manuscript for a detailed explanation of each method. Peak positions in cm^{-1}. "A" stands for Absent. Numbers in parentheses indicate the relative error.

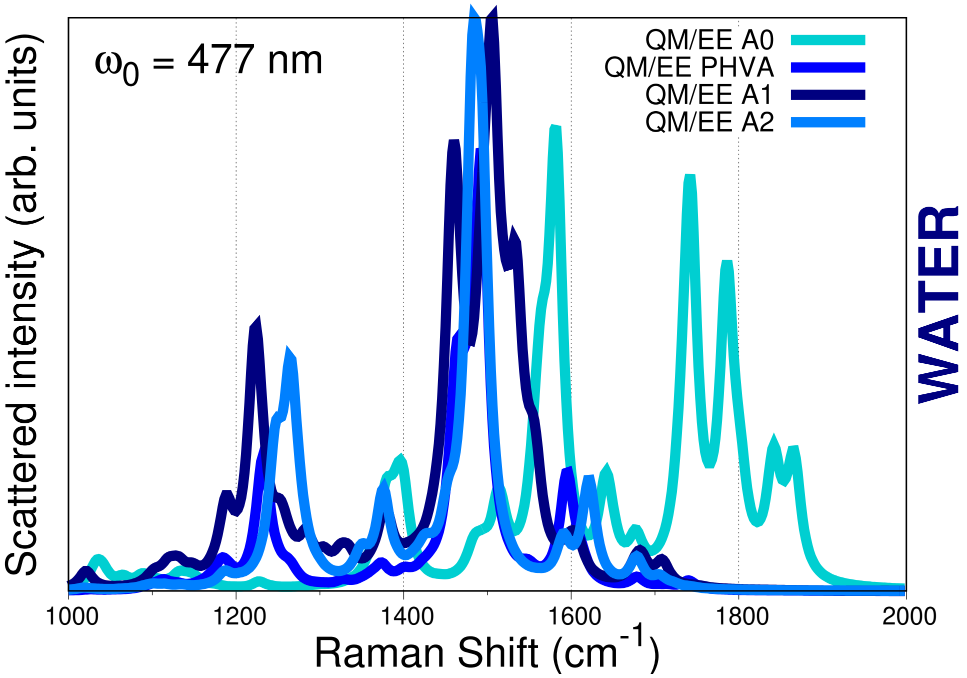

Figure S7: Comparison between the QM/EE spectra of DOX in water, after applying A0, PHVA, A1, and A2 strategies on the ten representative structures

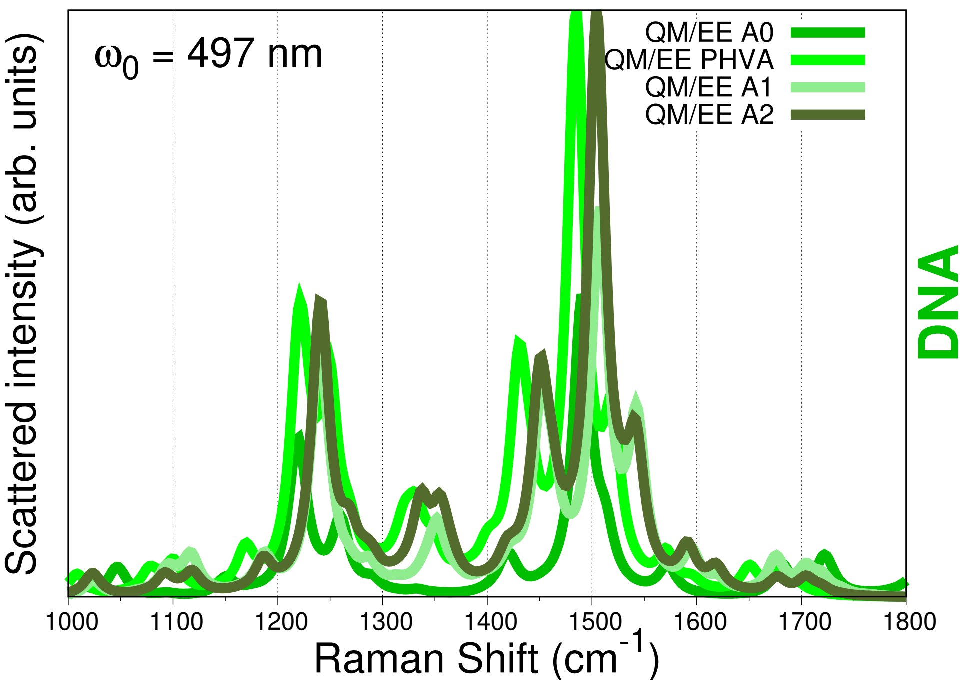

Figure S8: Comparison between the QM/EE spectra of DOX in DNA solutions, after applying A0, PHVA, A1, and A2 strategies on the six representative structures

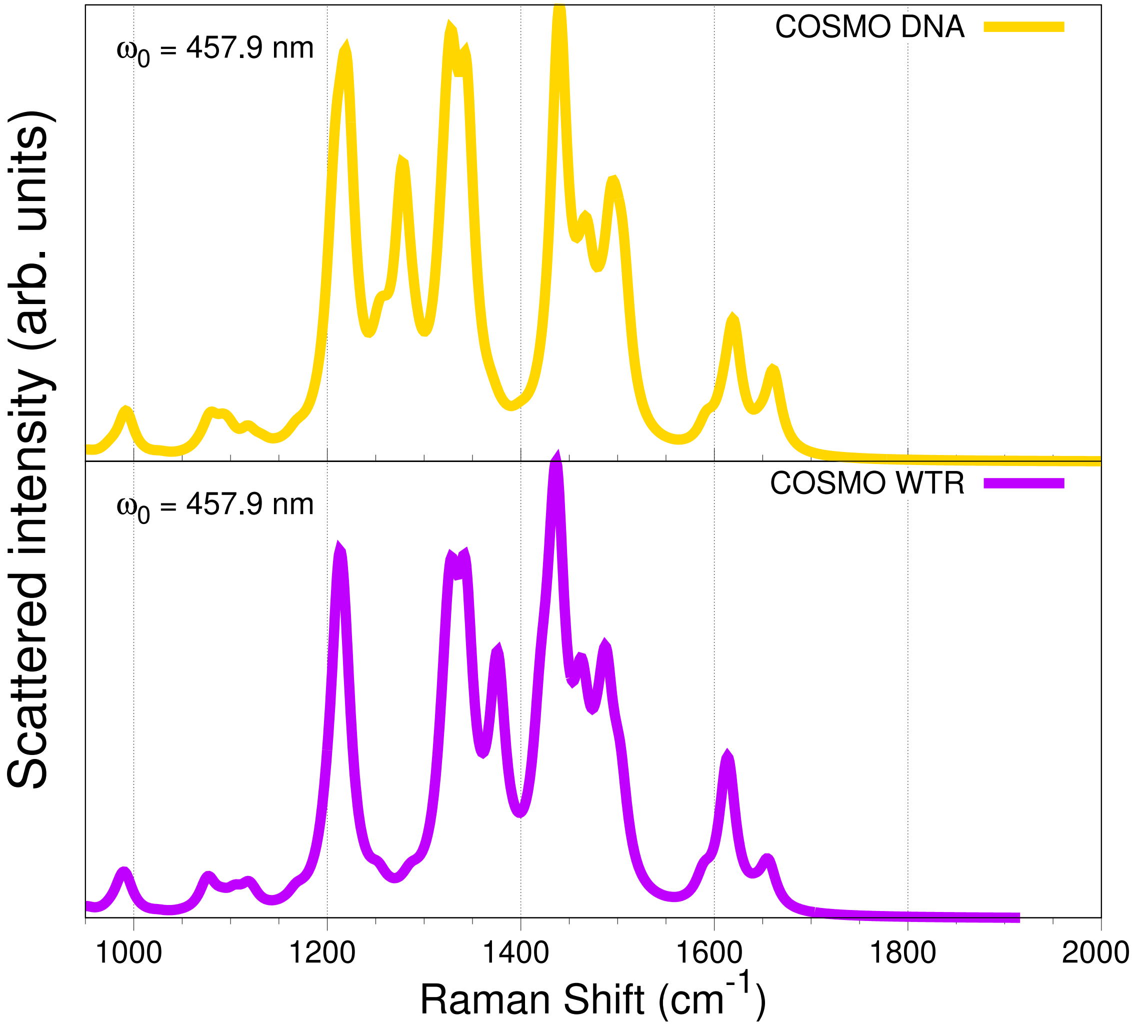

Figure S9: Comparison between RR spectra of DOX in water and in DNA, treating the environment as a structureless continuum, characterized by its dielectric constant.

References

[1] Nakata, Y.; Hopfinger, A. Predicted mode of intercalation of doxorubicin with dinucleotide dimers. *Biochem. Biophys. Res. Commun.* **1980**, *95*, 583–588.

[2] Barone, G.; Guerra, C. F.; Gambino, N.; Silvestri, A.; Lauria, A.; Almerico, A. M.; Bickelhaupt, F. M. Intercalation of Daunomycin into Stacked DNA Base Pairs. DFT Study of an Anticancer Drug. *J. Biomol. Struct. Dyn.* **2008**, *26*, 115–129, PMID: 18533732.

[3] Zhu, S.; Yan, L.; Ji, X.; Lu, W. Conformational diversity of anthracycline anticancer antibiotics: A density functional theory calculation. *J. Mol. Struct.: THEOCHEM* **2010**, *951*, 60–68.

[4] Lei, H.; Wang, X.; Wu, C. Early stage intercalation of doxorubicin to DNA fragments observed in molecular dynamics binding simulations. *J. Mol. Graph.* **2012**, *38*, 279–289.

[5] Airoldi, M.; Barone, G.; Gennaro, G.; Giuliani, A. M.; Giustini, M. Interaction of doxorubicin with polynucleotides. A spectroscopic study. *Biochemistry* **2014**, *53*, 2197–2207.

[6] Poudel, L.; Wen, A. M.; French, R. H.; Parsegian, V. A.; Podgornik, R.; Steinmetz, N. F.; Ching, W.-Y. Electronic structure and partial charge distribution of doxorubicin in different molecular environments. *ChemPhysChem* **2015**, *16*, 1451–1460.

[7] Jain, M.; Barthwal, S. K.; Barthwal, R.; Govil, G. Restrained molecular dynamics studies on complex of adriamycin with DNA hexamer sequence d-CGATCG. *Arch. Biochem.* **2005**, *439*, 12–24.

[8] Barthwal, R.; Agrawal, P.; Tripathi, A.; Sharma, U.; Jagannathan, N.; Govil, G. Structural elucidation of 4'-epiadriamycin by nuclear magnetic resonance spectroscopy and comparison with adriamycin and daunomycin using quantum mechanical and restrained molecular dynamics approach. *Arch. Biochem.* **2008**, *474*, 48–64.

[9] Agrawal, P.; Barthwal, S. K.; Barthwal, R. Studies on self-aggregation of anthracycline drugs by restrained molecular dynamics approach using nuclear magnetic resonance spectroscopy supported by absorption, fluorescence, diffusion ordered spectroscopy and mass spectrometry. *Eur. J. Med. Chem.* **2009**, *44*, 1437–1451.

[10] Jawad, B.; Poudel, L.; Podgornik, R.; Steinmetz, N. F.; Ching, W.-Y. Molecular mechanism and binding free energy of doxorubicin intercalation in DNA. *Phys. Chem. Chem. Phys.* **2019**, *21*, 3877–3893.

[11] Jawad, B.; Poudel, L.; Podgornik, R.; Ching, W.-Y. Thermodynamic Dissection of the Intercalation Binding Process of Doxorubicin to dsDNA with Implications of Ionic and Solvent Effects. *J. Phys. Chem. B* **2020**, *124*, 7803–7818, PMID: 32786213.

[12] Lafiosca, P.; Gómez, S.; Giovannini, T.; Cappelli, C. Absorption Properties of Large Complex Molecular Systems: The DFTB/Fluctuating Charge Approach. *J. Chem. Theory Comput.* **2022**, *18*, 1765–1779.

[13] Rodrigues, E. S. B.; Macêdo, I. Y. L. d.; Silva, G. N. d. M. e.; de Carvalho e Silva, A.; Gil, H. P. V.; Neves, B. J.; Gil, E. d. S. DNA-Based Electrodes and Computational Approaches on the Intercalation Study of Antitumoral Drugs. *Molecules* **2021**, *26*, 7623.

[14] Hillig, K. W.; Morris, M. D. Pre-resonance Raman spectra of adriamycin. *Biochem. Biophys. Res. Commun.* **1976**, *71*, 1228–1233.

[15] Manfait, M.; Bernard, L.; Theophanides, T. Resonance and pre-resonance Raman spectra of the antitumor drugs adriamycin and daunomycin. *J. Raman Spectrosc.* **1981**, *11*, 68–74.

[16] Manfait, M.; Alix, A. J.; Jeannesson, P.; Jardillier, J.-C.; Theophanides, T. Interaction of adriamycin with DNA as studied by resonance Raman spectroscopy. *Nucleic Acids Res.* **1982**, *10*, 3803–3816.

[17] Angeloni, L.; Smulevich, G.; Marzocchi, M. Absorption, fluorescence and resonance Raman spectra of adriamycin and its complex with DNA. *Spectrochimica Acta Part A: Molecular Spectroscopy* **1982**, *38*, 213–217.

[18] Manfait, M.; Theophanides, T. Fourier Transform Infrared Spectra of cells treated with the drug adriamycin. *Biochem. Biophys. Res. Commun.* **1983**, *116*, 321–326.

[19] Smulevich, G.; Feis, A. Surface-enhanced resonance Raman spectra of adriamycin, 11-deoxycarminomycin, their model chromophores, and their complexes with DNA. *J. Phys. Chem.* **1986**, *90*, 6388–6392.

[20] Nonaka, Y.; Tsuboi, M.; Nakamoto, K. Comparative study of aclacinomycin versus adriamycin by means of resonance Raman spectroscopy. *Journal of Raman spectroscopy* **1990**, *21*, 133–141.

[21] Beljebbar, A.; Sockalingum, G.; Angiboust, J.; Manfait, M. Comparative FT SERS, resonance Raman and SERRS studies of doxorubicin and its complex with DNA. *Spectrochim. Acta A Mol. Biomol. Spectrosc.* **1995**, *51*, 2083–2090.

[22] Yan, Q.; Priebe, W.; Chaires, J. B.; Czernuszewicz, R. S. Interaction of doxorubicin and its derivatives with DNA: Elucidation by resonance Raman and surface-enhanced resonance Raman spectroscopy. *Biospectroscopy* **1997**, *3*, 307–316.

[23] Smulevich, G.; Mantini, A. R.; Feis, A.; Marzocchi, M. P. Resonance Raman spectra and transform analysis of anthracyclines and their complexes with DNA. *J. Raman Spectrosc.* **2001**, *32*, 565–578.

[24] Lee, C.-J.; Kang, J.-S.; Kim, M.-S.; Lee, K.-P.; Lee, M.-S. The study of doxorubicin and its complex with DNA by SERS and UV-resonance Raman spectroscopy. *Bull Korean Chem Soc.* **2004**, *25*, 1211–1216.

[25] Das, G.; Nicastri, A.; Coluccio, M. L.; Gentile, F.; Candeloro, P.; Cojoc, G.; Liberale, C.; De Angelis, F.; Di Fabrizio, E. FT-IR, Raman, RRS measurements and DFT calculation for doxorubicin. *Microsc. Res. Tech.* **2010**, *73*, 991–995.

[26] Szafraniec, E.; Majzner, K.; Farhane, Z.; Byrne, H. J.; Lukawska, M.; Oszczapowicz, I.; Chlopicki, S.; Baranska, M. Spectroscopic studies of anthracyclines: structural characterization and in vitro tracking. *Spectrochim. Acta A Mol. Biomol. Spectrosc.* **2016**, *169*, 152–160.

[27] Zhang, X.; Poniewierski, A.; Sozański, K.; Zhou, Y.; Brzozowska-Elliott, A.; Holyst, R. Fluorescence correlation spectroscopy for multiple-site equilibrium binding: a case of doxorubicin–DNA interaction. *Phys. Chem. Chem. Phys.* **2019**, *21*, 1572–1577.
